# Quantitative assessment of landslide hazard and risk at regional-scale: a case study from central Vietnam

**DOI:** 10.1007/s11356-025-37189-3

**Published:** 2025-12-18

**Authors:** Raja Das, Pham Van Tien, Karl W. Wegmann

**Affiliations:** 1 Center for Geospatial Analytics, North Carolina State University, 2800 Faucette Dr, Raleigh, NC 27695 USA; 2 Institute of Earth Sciences, Vietnam Academy of Science and Technology, No. 68 Huynh Thuc Khang Street, Hanoi, Vietnam; 3 Department of Marine, Earth, and Atmospheric Sciences, North Carolina State University, 2800 Faucette Dr, Raleigh, NC 27695 USA

**Keywords:** Landslide susceptibility, Machine learning, Probabilistic landslide hazard, Landslide risk, Index of connectivity (IC)

## Abstract

Effective management of landslide hazards depends on the ability to accurately predict their location, size, and timing, enabling effective strategies to mitigate their impact, safeguard communities, and reduce societal risks. This study details a comprehensive probabilistic landslide hazard assessment at a regional scale in central Vietnam, analyzing spatial, temporal, and magnitude probabilities of landslides and proposes a methodology for quantitative landslide risk assessment. This study utilized a total of 21,234 landslide occurrences from three inventories created from Typhoon Ketsana (2009), Tropical Storm Podul (2013), and Typhoon Molave (2020) for the landslide hazard and risk assessment. The landslide spatial probability was delineated for three landslide size classes ($100\ge {m}^{2}$, $1000\ge {m}^{2}$, and $\mathrm{10,000}\ge {m}^{2}$) by developing landslide susceptibility models using nine geo-factors and a Random Forest machine learning algorithm. The temporal landslide probability at the district level was determined by integrating recurrence intervals, calculated from the past six years’ landslide incidences, into a Poisson model to estimate the likelihood of experiencing one or more landslides at a location in the next ten years. Landslide size probabilities were derived from frequency–area distributions of the landslides from the inventories. The landslide hazard model was developed by synthesizing spatial, temporal, and size probabilities—presuming their independence. Nine landslide hazard scenarios were developed considering three time frames (2, 5, and 10 years) and three landslide size categories. Finally, the landslide risk models for streams and roads over the next 2-, 5-, and 10 years were computed by integrating the spatial, temporal, and size probabilities of landslides with the index of connectivity (IC). Landslide risk models indicate increased landslide risk for road networks and streams (hydropower infrastructure) for longer periods.

**Supplementary Information:**

The online version contains supplementary material available at 10.1007/s11356-025-37189-3.

## Introduction

Landslides are a major geo-hazard in mountainous regions, annually causing thousands of casualties and billions of dollars of property loss worldwide. Regions affected by recurrent devastating landslides necessitate thorough scientific investigation that facilitates predictions of these events regarding their spatial, temporal, and magnitude dimensions to enhance understanding of the phenomena and formulate effective mitigation strategies. The landslide hazard is defined as the likelihood of a landslide of a given magnitude in a given time and space (Varnes 1984; Guzzetti et al. 1999). Prediction of spatial occurrences of landslides, otherwise known as landslide susceptibility mapping, enables delineation of future landslide zones. However, it cannot provide further insights into’when’ and’how big’ the future landslides would be. Therefore, it is important to go beyond the spatial prediction of landslides and estimate the frequency and magnitude of landslides, and integrate landslide prediction capabilities concerning”where,” when,” and”magnitude” is necessary to address landslide hazards effectively (e.g., Van Westen et al. 2006; Guzzetti et al. 2005; Ghosh et al. 2009). Quantitative landslide hazard estimation considers the joint probability of spatial, temporal, and size magnitude of landslides, assuming these three probabilities are mutually independent (Guzzetti et al. 2005; Jaiswal et al. 2010).

Effective landslide hazard assessment requires integrating these spatial, temporal, and magnitude probabilities to capture where, when, and how large landslides may occur. Spatial probability is commonly derived through susceptibility mapping and often modeled using logistic regression or machine learning based on historical events and terrain attributes (Ghosh et al. 2012; Bui et al. 2013; Arrogante-Funes et al. 2021; Das et al. 2024). Temporal probability, typically estimated from landslide frequencies or rainfall thresholds, is essential in monsoon-prone regions (Bui et al. 2013; Yang et al. 2020). Magnitude probability, derived from volume or area–frequency relationships, estimates the likely scale and impact of events (Guzzetti et al. 2005; Ghosh et al. 2012).

Landslide hazard is thus best characterized by the joint probability of spatial occurrence, temporal frequency, and magnitude. This integration enables the generation of probabilistic hazard scenarios that reflect the likelihood of landslides of a given size occurring in a specific location within a defined period. Recent methodological innovations have enabled the practical implementation of this integrated framework. Das et al. (2011) contributed to this shift by introducing the concept of Homogeneous Susceptible Units (HSUs), which improved the spatial delineation of hazard-prone areas by grouping terrain segments with similar landslide susceptibility characteristics. Jaiswal et al. (2010) further advanced the integrated hazard modeling approach by developing probabilistic scenarios that linked landslide volumes and temporal recurrence to infrastructure vulnerability in southern India. At the community level, Fu et al. (2020) also implemented this approach in western Hubei, China, by combining all three hazard components in a community-scale model. Additional applications of this integrated approach include the work of Ghosh et al. (2012), who applied spatial–temporal–magnitude modeling to assess landslide risk in the Darjeeling Himalayas using a frequency–area relationship. Similarly, Zêzere et al. (2004) developed a basin-scale probabilistic model incorporating spatial data and return period analysis in Portugal. These studies highlight a growing trend toward comprehensive, probabilistic models that improve our ability to anticipate and manage landslide hazards across diverse spatial scales and planning contexts.

A landslide risk assessment considers the expected damage or loss incurred due to the landslide hazard (Varnes 1984; Van Westen et al. 2006). The landslide risk model integrates information on socially vulnerable elements to landslides, such as buildings, roads, and other infrastructure, with the landslide hazard model to quantify the potential economic losses and human casualties due to landslides (Hervás and Bobrowsky 2009). However, performing a quantitative landslide hazard assessment and risk analysis requires a substantial coverage of historical landslide records, which are often not readily available, especially in developing countries. Incompleteness in the historical landslide records is a serious deterrent in predicting landslide hazard scenarios, hindering subsequent risk analysis. Considering the absence of substantial historical landslide records coupled with the lack of a universal standardized accepted methodology for computing landslide hazard and risk, many studies in the literature have used the terminologies ‘susceptibility,’ ‘hazard,’ and ‘risk’ interchangeably. In such cases, landslide susceptibility models have largely been conceptualized as landslide hazard or landslide risk models (Metternicht et al. 2005; Aksha et al. 2020; Banshtu et al. 2020; Saleem et al. 2020; Ram and Gupta 2021). This situation is very common, especially in developing countries, due to information asymmetry and the limited availability of utilitarian datasets accessible to GIS platform processing for landslide hazard and risk assessment. As a result, the volume of research explicitly focused on hazard and risk assessment remains relatively small compared to the extensive literature on landslide susceptibility modeling. Nevertheless, some recent studies have made notable progress in operationalizing risk frameworks by incorporating exposure and vulnerability into hazard assessments. For example, Fu et al. (2020) integrated population and asset exposure into their hazard model to estimate potential losses at the community level. Similar approaches in Bhutan (Dikshit et al., 2020) and Mexico (Arrogante-Funes et al., 2021) combined hazard probabilities with social and ecological vulnerability indicators to evaluate landslide risk in data-scarce environments.

The rising frequency of fatal landslides globally represents an alarming trend, particularly acute in the mountainous regions of tropical countries (Froude and Petley 2018; Jakob 2022). These areas are increasingly vulnerable to landslides, largely due to a surge in extreme precipitation events caused by intense typhoons and hurricanes (Jakob 2022). In Southeast Asia, Vietnam stands out as one of the nations most affected by landslides, with torrential rains from typhoons identified as the primary catalyst for these devastating events (Tien et al. 2016; Le Hong and Van 2016; Van Tien et al. 2021a, 2021b). The central region of Vietnam is especially susceptible to natural hazards such as landslides and extensive flooding, and landslides caused by extreme weather events have resulted in significant damage and casualties in the region over the past several decades (Tien et al. 2016; Duc et al. 2020; Van Tien et al. 2021a). Previous studies in this region have largely focused on computing the landslide susceptibility utilizing incomplete and non-time-stamped inventory data (Meinhardt et al. 2015; Bui et al. 2020). However, no attempts have been made to perform a probabilistic landslide hazard assessment and estimate subsequent risk due to landslides in the high landslide-prone region of central Vietnam. Therefore, this study aims to (1) Develop landslide susceptibility models based on landslide size distribution; (2) construct different probabilistic landslide hazard scenarios based on spatial, temporal, and size magnitude probabilities of landslides; (3) propose a novel framework for computing landslide risk suitable for data-sparse locations.

## Study area

**Fig. 1:**
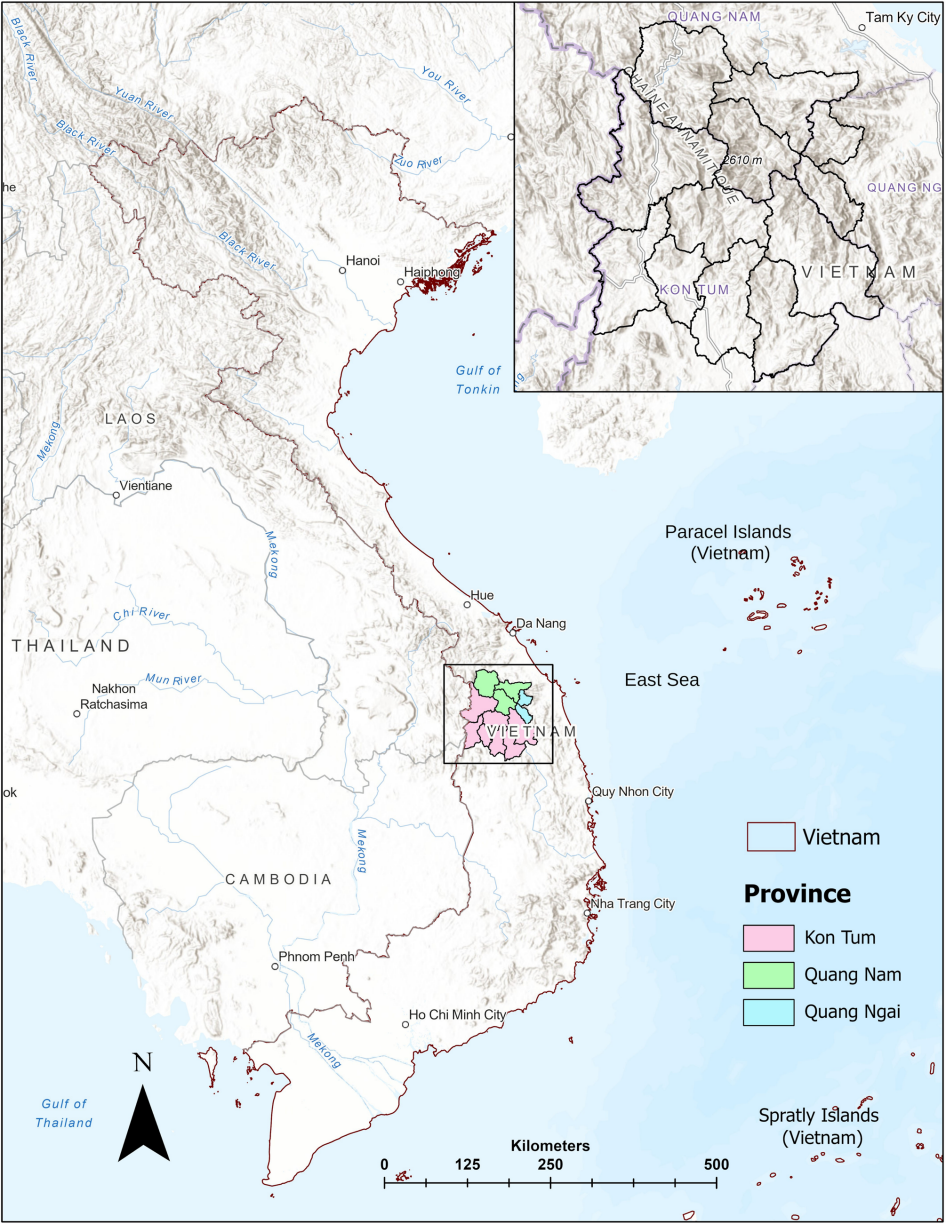
Geographic map of Vietnam highlighting the study area. The administrative boundaries of the three different provinces are marked in distinct colors. The inset map shows the districts overlying the regional topography

The study area encompasses twelve administrative districts within three provinces in central Vietnam and covers an area of 10,409 km^2^. This includes seven districts in Kon Tum province within the Central Highland region, which constitute 66% of the study area, along with three districts from southern Quang Nam and two from western Quang Ngai in the South-Central Coast region (Fig. 1). The terrain is marked by steep mountains and deep valleys, with altitudes ranging from 29 to 2,588 m above sea level, and a prominent high mountain range extending from the northeast to the southwest over 135 km. The landscape’s elevation decreases towards the north and east, particularly in Quang Nam and Quang Ngai provinces, transitioning into flatter valleys and plains towards the south. The region is characterized by significant rainfall, with variations in the timing and length of the rainy season across the provinces. Kon Tum experiences annual rainfall between 1,700 and over 3,000 mm, with the rainy season from April to November. In Quang Nam, rainfall averages between 2,020 and 4,000 mm annually, from September to December, while Quang Ngai sees 2,500 to 3,500 mm, typically from August to November (Hùng and Dũng 2013; Bui et al. 2020; Duc et al. 2020; Nguyen et al. 2023). Geologically, the area is primarily underpinned by the Kon Tum Massif (KTM), the largest exposed Precambrian basement in the Indochina block, consisting of Mesoproterozoic igneous and late Paleoproterozoic to late Neoproterozoic sedimentary formations (Fangqian et al. 2020; Nakano et al. 2021). The KTM is dissected by prominent fault systems, including the Po Ko fault (north–south trending) and the Tam Ky–Phuoc Son fault zone (east–west trending), the latter of which is considered an ancient suture between the massif and the Truong Son Belt (Nakano et al. 2021). The study area is subdivided into three major tectonic formations (Tac Po, Kham Duc, and Song Re) and several complexes, such as Ba Na, Ben Giang-Que Son, and Hai Van, are distinguished by lithology and metamorphic grade. The KTM’s metasedimentary rocks show a wide range of metamorphic grades, from low-grade schists to high-grade granulites, and include granite gneiss and migmatite (Osanai et al. 2004). Additionally, the region hosts Neogene to Quaternary sedimentary deposits, including conglomerates, sandstones, and siltstones, particularly around Kon Tum region (Carter et al. 2000). The study area is also dissected by major fault systems like Tam Ky-Phuoc Son fault zone, Hung Nhuong-Ta Vi fault zone, the Po Ko and Dac To Kan faults, along with numerous smaller ones, contributing to the area’s susceptibility to mass wasting phenomena (Jiang et al. 2022).

## Materials and methods

### Landslide data

**Fig. 2:**
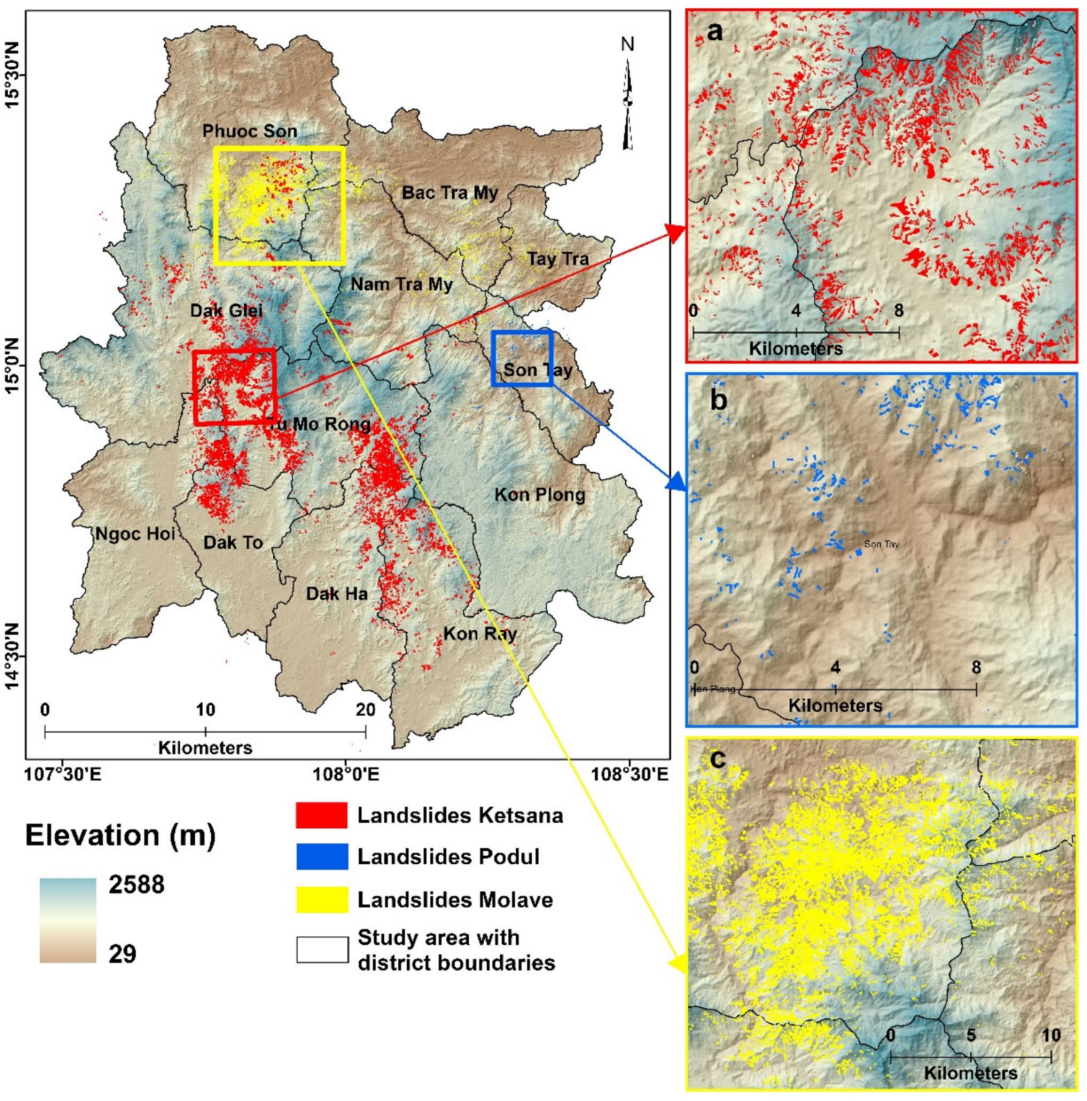
Map showing the location of landslides triggered by (**a**) Ketsana, (**b**) Podul, and (**c**) Molave marked in red, blue, and yellow colors, respectively

Landslides triggered by Typhoon Ketsana (2009), Tropical Storm Podul (2013), and Typhoon Molave (2020) were identified from high-resolution satellite imagery (Fig. 2) (Das et al. 2024). RapidEye satellite imagery (5 m/px) was used for mapping landslides of Ketsana and Podul, while PlanetScope (3 m/px) was used for Molave. The differential Normalized Difference Vegetation Index (dNDVI) was calculated from pre and post landslide event imagery to initially detect landslides triggered by Tropical Storm Podul and Typhoon Molave. The dNDVI map indicates changes in vegetation cover due to landslides and concurrent flooding, and landslide zones were manually identified and mapped from the dNDVI map. Without any pre-event images for Typhoon Ketsana, landslides were identified by applying various NDVI thresholds to differentiate landslides from other objects on the image, which was then manually refined to separate and map landslides. Analysis-ready satellite images were collected with the lowest possible cloud cover and shortest temporal acquisition time for mapping the landslides. A total of 8,744 landslides were mapped for Typhoon Ketsana, having a cumulative area of 51.39 km^2^ with a mean landslide size of 5,877 m^2^. In contrast, Tropical Storm Podul triggered fewer landslides, totaling 915, with a cumulative landslide area of 1.42 km^2^ and a mean size of 1,556 m^2^. Typhoon Molave triggered a total of 11,575 landslides, encompassing a cumulative area of 36.66 km^2^ with a mean landslide size of 3,167 m^2^.

### Landslide size probability estimation

The mapped landslides’ size probability distribution was calculated for individual and cumulative landslide inventories in the study area. A probability density function (PDF) proposed by Malamud et al. (2004) was employed to calculate the probability density distribution (*p*) of a given-sized landslide in the inventories using the following formula:

1 $$p\left({A}_{L}\right)=\frac{1}{{N}_{L}}\frac{\updelta {N}_{L}}{\updelta {A}_{L}}$$

where ${A}_{L}$ is the landslide area, ${N}_{L}$ is the total number of landslides in an inventory, $\updelta {N}_{L}$ is the number of landslides with areas between ${A}_{L}$ and ${A}_{L}+\updelta {A}_{L}$, and $\updelta {A}_{L}$ is the bin width.

Subsequently, the cumulative probability (P) of landslide occurrence for a specific size was computed from the combined inventory for three landslide-triggering events. Determining P enables the calculation of the probability of a landslide having a size $\left({A}_{L}\right)$ greater than or equal to a minimum size, ${a}_{L},$ as:

2 $${P}_{{A}_{L}}=P\left[{A}_{L}\ge {a}_{L}\right]$$

### Landslide temporal probability estimation

A Poisson distribution model (Crovelli 2000; Coe et al. 2004; Guzzetti et al. 2005) was used to estimate the exceedance probability of landslide occurrences in time. According to the Poisson distribution model, the probability of occurrence of *n* landslides during time *t* is obtained from the equation:

3 $$P\left[{N}_{L}\left(t\right)=n\right]=\frac{\uplambda {t}^{n}}{n!}{e}^{-}\left(\uplambda t\right)$$

where $P\left[{N}_{L}\left(t\right)=n\right]$ is the exceedance probability of occurrence of n number of landslides (n = 0, 1, 2,…) in the period t, while λ is the average rate of landslide occurrence. It is the reciprocal of the mean recurrence interval (µ), i.e., µ = 1/λ, calculated from the historical record of landslides occurrences. Subsequently, using (Eq. 3), the exceedance probability of one or more landslides during time *t* was calculated as follows:

4 $$P\left[{N}_{L}\left(t\right)\ge 1\right]=1-P\left[{N}_{L}\left(t\right)=0\right]=1-{e}^{-}\left(\uplambda t\right)=1-{e}^{-}\left(t/\upmu \right)$$

In order to capture the spatio-temporal heterogeneity of landslide occurrences, the mean recurrence interval or return period was estimated for each district in the study area using available landslide records from the past six years (2018–2023). Subsequently, the Poisson probability model was employed to compute the exceedance probability of one or more landslides for 1 to 10 years. Since a complete spatio-temporal landslide inventory is not available or only partially available in the study area and given the sparse nature of available landslide information prior to 2018, the recurrence interval estimation was restricted to the last six years of data. Incorporating landslide information from before 2018 would have led to underestimating the number of landslides and inflation of the return period, resulting in erroneous results. The last six years of landslide records provide a reliable and accurate spatio-temporal coverage of landslides and is a representative sample of the current temporal trends of landslides in the region, thus facilitating the calculation of accurate future temporal probability of landslides.

### Landslide susceptibility analysis

The study utilized nine explanatory variables or geo-factors for the development of landslide susceptibility models, namely, elevation, slope angle, aspect, Topographic Position Index (TPI), Topographic Wetness Index (TWI), distance to fault, drainage density, lithology, and annual average rainfall (Supplementary Fig. B.2). A 30-m NASADEM (NASA JPL 2020), an improved product of Shuttle Radar Topography Mission (SRTM) digital elevation model, was utilized to generate the topographic variables. Annual precipitation data was derived from the Climate Hazards Group InfraRed Precipitation with Station data (CHIRPS) (Funk et al. 2015) from 1990 to 2020 in the rainy season between July and November when most of the landslide activities take place in the region. The lithological and fault maps were derived from Vietnam’s 1:200,000-scale Geological and Mineral Resources map. The geology was reclassified into seven groups based on the lithological composition, relative weathering rate, and strength parameters (Supplementary Table B.1). The Random Forest algorithm was utilized to construct susceptibility models, detailed in Appendix A.1 of the supplementary materials. Three distinct landslide susceptibility models were developed, reflecting the size variations of landslides cataloged in the Ketsana, Podul, and Molave events inventories. Landslides were classified into three size categories: $100\ge {m}^{2}$, $1000\ge {m}^{2}$, and $\mathrm{10,000}\ge {m}^{2}$ for developing the susceptibility models. Each size category was divided into training and validation subsets with a 75:25 split. Points representing landslides were generated randomly within the defined polygons, whereas non-landslide points were placed at a minimum distance of 100 m from any landslide polygon. The execution of the Random Forest models was conducted using R Studio statistical software.

### Index of connectivity development

Landslides can inflict significant damage on infrastructure, settlements, and other objects if the landslide initiation zone, the travel path of landslide debris, and the target objects are topographically connected. The transportation of sediment generated through landslides can only occur if connected through topographic pathways or channels. Thus, only those landslides connected topographically with targets pose a substantial threat of causing destruction. Consequently, understanding such connectivity is crucial in delineating landslide risk zones and formulating effective mitigation strategies. The index of connectivity (IC) of a landscape, initially defined by Borselli et al. (2008), provides a quantitative measure of the topographic connectivity of an object. IC represents the logarithmic ratio of the upslope contributing area for sediment production and downslope sediment flux path length to the nearest target (See Appendix A.2 for detail). In landslide risk assessment, the connectivity between the topography and the elements at risk, such as roads, buildings, etc., can be effectively delineated. If a well-connected part of the topography affiliated with an asset is prone to landslides, the asset will be at high risk due to landslides. The value of IC ranges from + ∞ to -∞, a higher IC indicating greater connectivity between the source and target locations, and vice versa. The study computed the IC for road corridors (IC_Road_) and river channels (IC _River_) using the method proposed by (Cavalli et al. 2013) and implemented in the standalone version of SedInConnect (https://github.com/HydrogeomorphologyTools), developed by Crema and Cavalli (2018). The 30-m NASADEM was utilized to develop the IC models since higher-resolution digital elevation models are unavailable for the study area. Appendix A.2. in the Supplementary material describes the working formula for computing IC models.

### Landslide hazard and risk estimation

A probabilistic landslide hazard assessment considers (i) magnitude probability (P_M_), (ii) temporal probability (P_T_), and (iii) spatial probability (P_S_) of landslide occurrences, assuming that these probabilities are mutually independent and landslide hazard consists of the joint probability of these three probabilities; hence, landslide hazard (L_H_) can be expressed as:

5 $${L}_{H}={P}_{M}\times {P}_{T}\times {P}_{S}$$

Landslide size is a proxy for landslide magnitude in this study. On the other hand, landslide risk can be understood as the potential impact or consequences of a landslide in terms of injuries or lives lost and infrastructure/property damage (Varnes 1984). This study conceptualizes landslide risk as the function of the spatio-temporal probability of landslides and the associated index of connectivity of the landscape between sediment sources (hillslopes) and receiving areas (channels). Besides the spatial scale, the probability of landslide occurrence of different sizes also varies over time. Hence, landslide risk was quantified by integrating the landslide hazard probabilities with the normalized IC (on a scale of 0–1). A higher value indicates a greater risk of landslide-related damage to an object. The study’s landslide risk assessment assumes that areas with high landslide hazard zones are more likely to experience landslides, and objects connected to high landslide hazard zones through high IC values are at increased risk of landslide-related damage. Considering the spatial, temporal, and size probabilities of landslides with topographic connectivity provides a more comprehensive understanding of landslide risk that can inform decision-making for risk reduction and management.

## Results

### Landslide size probability estimation

**Fig. 3:**
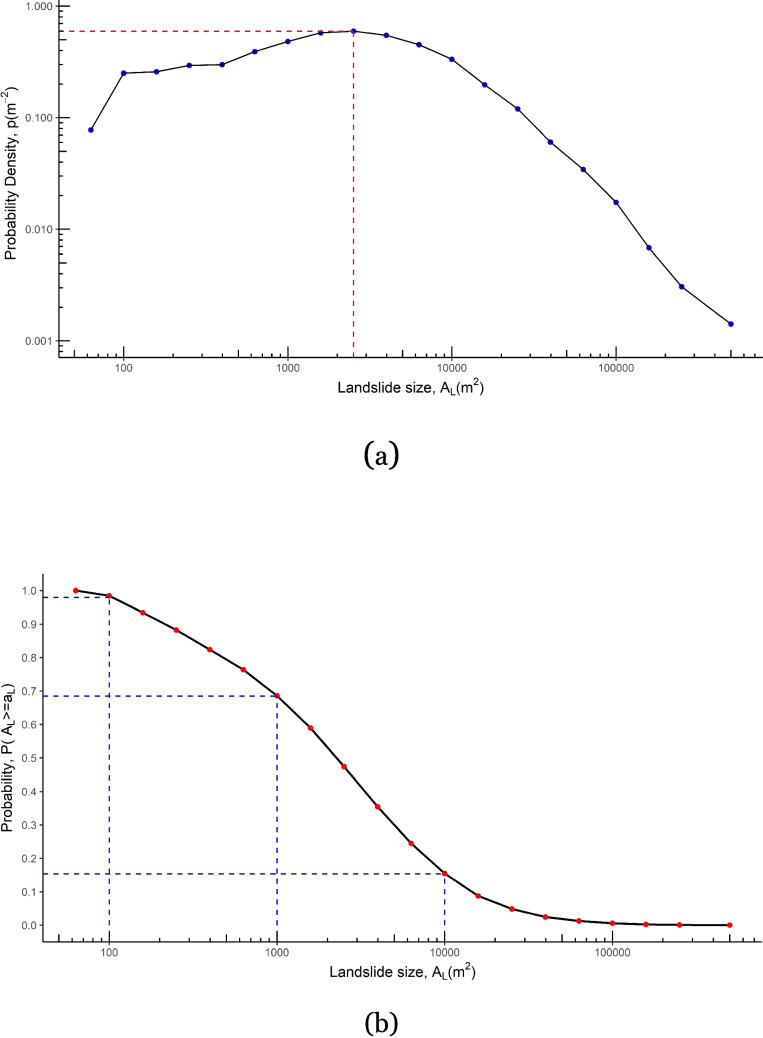
Probability density distribution (**a**) and cumulative probability (b) of landslide size computed from landslide inventories of three major landslide-triggering events in the study area

The probability density function (PDF) was computed for each of the three individual landslide inventories, as well as for the cumulative inventories, to analyze the landslide size-density distribution and to identify the rollover points where the negative power-law decay of the landslide size-frequency distribution begins (Malamud et al. 2004; Van Den Eeckhaut et al. 2007). Beyond the rollover point, the probability density of landslides decreases with increasing landslide area. Figure 3a shows the rollover point of the landslide areas calculated at 0.20 bin width for the combined inventories of Ketsana, Podul, and Molave-triggered landslides, which is about 2,511 m^2^. A cumulative probability distribution of landslide size was computed to estimate the probability of a landslide exceeding a certain size in the landslide inventories. The estimated probabilities of landslide sizes exceeding 100 m^2^, 1,000 m^2^, and 10,000 m^2^ in the study area were 0.98, 0.68, and 0.15, respectively, calculated by combining landslide size from all three inventories (Fig. 3b). These values indicate that there is a 98%, 68% and 15% probability of a landslide size exceeding 100 m^2^, 1,000 m^2^, and 10,000 m^2^, respectively, based on the analysis of the three most recent and prominent landslide-triggering events in the study area.

### Temporal probability of landslides

The recurrence interval or return period of landslides, i.e., the average expected time between two consecutive failures, was calculated for each district based on the total number of landslide events between 2018 and 2023. Assuming that the last six years of landslide occurrences are representative of the current landslide recurrence in individual districts and will remain constant in the future, the Poisson model (Eq. 3) was adapted to compute the exceedance probability of one or more landslides occurring in each of the next ten years for all study area districts (Table 1).

**Table 1:** The exceedance probability of landslides occurrence in different years across the districts computed from the Poisson model

District	1 year	2 years	3 years	4 years	5 years	6 years	7 years	8 years	9 years	10 years
Dak Glei	0.63	0.86	0.95	0.98	0.99	1	1	1	1	1
Dak Ha	0.57	0.81	0.92	0.96	0.98	0.99	1	1	1	1
Dak To	0.49	0.74	0.86	0.93	0.96	0.98	0.99	1	1	1
Kon Plong	0.78	0.95	0.99	1	1	1	1	1	1	1
Kon Ray	0.63	0.86	0.95	0.98	0.99	1	1	1	1	1
Ngoc Hoi	0.49	0.74	0.86	0.93	0.96	0.98	0.99	1	1	1
Tu Mo Rong	0.63	0.86	0.95	0.98	0.99	1	1	1	1	1
Bac Tra My	0.92	0.99	1	1	1	1	1	1	1	1
Nam Tra My	0.94	1	1	1	1	1	1	1	1	1
Phuoc Son	0.89	0.99	1	1	1	1	1	1	1	1
Son Tay	0.86	0.98	1	1	1	1	1	1	1	1
Tay Tra	0.69	0.9	0.97	0.99	1	1	1	1	1	1

The probability of landslides occurring in one year varies across the study area, ranging between 0.49 (49%) in Dak To and Ngoc Hoi districts of Kon Tum province and 0.94 (94%) in Nam Tra My district of Quang Nam province. Generally, the temporal probability of landslides is very high in the Bac Tra My, Nam Tra My, and Phuoc Son districts of Quang Nam province, high in the Son Tay and Tay Tra districts of Quang Ngai province, and mostly moderate (less than 65%) in the Dak Glei, Dak Ha, Dak To, Kon Ray, Ngoc Hoi, and Tu Mo Rong districts of Kon Tum province for one year. However, the probability of one or more landslides increases over time, with all districts exceeding a 90% probability of landslides occurring in the next four years and five districts reaching a percent probability of landslide occurrence during this time. It is reasonable to expect that the temporal probability of at least one landslide event occurring is extremely high across the districts in the next five years, with all districts exceeding 95% probability, among which six districts attain 100% landslide occurrence probability in this period. We consider the exceedance probabilities for two, five, and ten years to construct various landslide hazard scenarios.

### Spatial probability of landslides

**Fig. 4:**
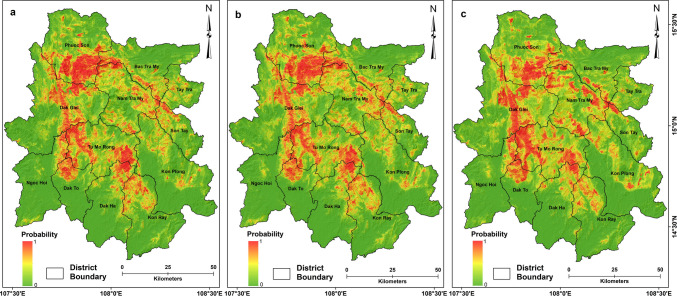
Landslide susceptibility maps of the study area developed using the Random Forest algorithm for landslide size (**a**) ≥ 100 m^2^, (**b**) ≥ 1,000 m^2^, and (**c**) ≥ 10,000 m^2^

Three landslide susceptibility models were generated for landslide sizes ≥ 100 m^2^, ≥ 1000 m^2^, and ≥ 10,000 m^2^ in the study area (Fig. 4a-c). The landslide susceptibility model for landslide size ≥ 100 m^2^ classified 9.83% and 10.32% study as very highly and highly, respectively, and 48.03% are classified as very low susceptible zones for landslides (Fig. 4a). The landslide susceptibility model for landslide size ≥ 1,000 m^2^ classified 45.69% of the area as very low, 11.2% as high, and 10.39% as very high (Fig. 4b). The model for landslide size ≥ 10,000 m^2^ categorized 44.66% of the area as very low, 10.95% as high, and 9.96% as very high in landslide susceptibility (Fig. 4c). Districts such as Phuoc Son, Nam Tra My, and Tu Mo Rong remain very highly susceptible, and Bac Tra My, Son Tay, Dak Ha, the central part of Dak Glei, and northern parts of Kon Ray districts are highly susceptible to landslides of all sizes. In contrast, the southern and south-western parts of the study area are mostly low to very low in susceptibility for all sized landslides. This includes southern parts of Dak Glei, Dak To, Dak Ha, Kon Ray, and Ngoc Hoi districts, probably owing to their lower topographic altitude and relatively gentle slopes unfavorable for landslide initiation.

**Fig. 5:**
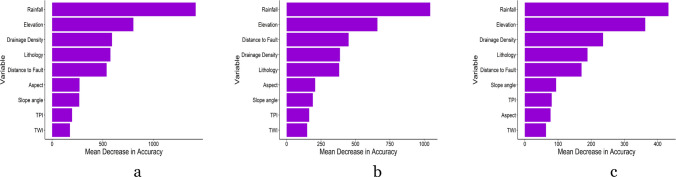
Plots showing Mean Decrease in Accuracy of the three landslide susceptibility models developed using the Random Forest algorithm for the (**a**) landslide size ≥ 100 m^2^, (**b**) landslide size ≥ 1,000 m^2^, and (**c**) landslide size ≥ 10,000 m^2^

Figures 5a-c depict the Mean Decrease in Accuracy (MDA) for the three landslide susceptibility models. MDA quantifies the model’s accuracy reduction when a variable is omitted. A higher decrease in accuracy indicates greater importance of the variable in achieving precise predictions. Rainfall and elevation consistently emerged as the two most crucial variables in the landslide susceptibility models across all landslide sizes. Additionally, drainage density, lithology, and distance to faults were identified as significant variables in the models, making them the top five important variables.

Table 2 illustrates the performance of the three landslide susceptibility models developed in this study. Out-of-Bag (OBB) errors were obtained during the development of the Random Forest models on training data, which are 6.62%, 7.48%, and 7.44% for susceptibility models of landslide size ≥ 100 m^2^, ≥ 1,000 m^2^, and ≥ 10,000 m^2^, respectively. Validation of the test data models yielded high accuracy for all models, which are 90.29%, 88.54%, and 86.98% for the landslide sizes ≥ 100 m^2^, ≥ 1,000 m^2^, and ≥ 10,000 m^2^, respectively. The sensitivity values of all susceptibility models are greater than 85%. The Area Under the Receiver Operating Characteristic Curve (AUC) was calculated, resulting in AUC values of 94.4%, 95.3%, and 96.6% for the models of landslide size ≥ 100 m^2^, ≥ 1,000 m^2^, and ≥ 10,000 m^2^, respectively (Supplementary Fig. C.3). The models’ high AUC values highlight their exceptional capacity in distinguishing between landslide and non-landslide areas, affirming their prediction accuracy and reliability.

**Table 2:** Out-of-bag (OBB) error rates of the Random Forest models in training data and other performance evaluation metrics calculated for the test data

Susceptibility Model	Out-of-bag error rate (%)	Accuracy (%)	Sensitivity (%)	Specificity (%)
Landslide size ≥ 100 *m*^2^	6.62	90.29	89.75	90.86
Landslide size ≥ 1,000 *m*^2^	7.48	88.54	88	89.08
Landslide size ≥ 10,000 *m*^2^	7.44	86.98	85.71	88.24

### Index of connectivity (IC) for roads and streams

**Fig. 6:**
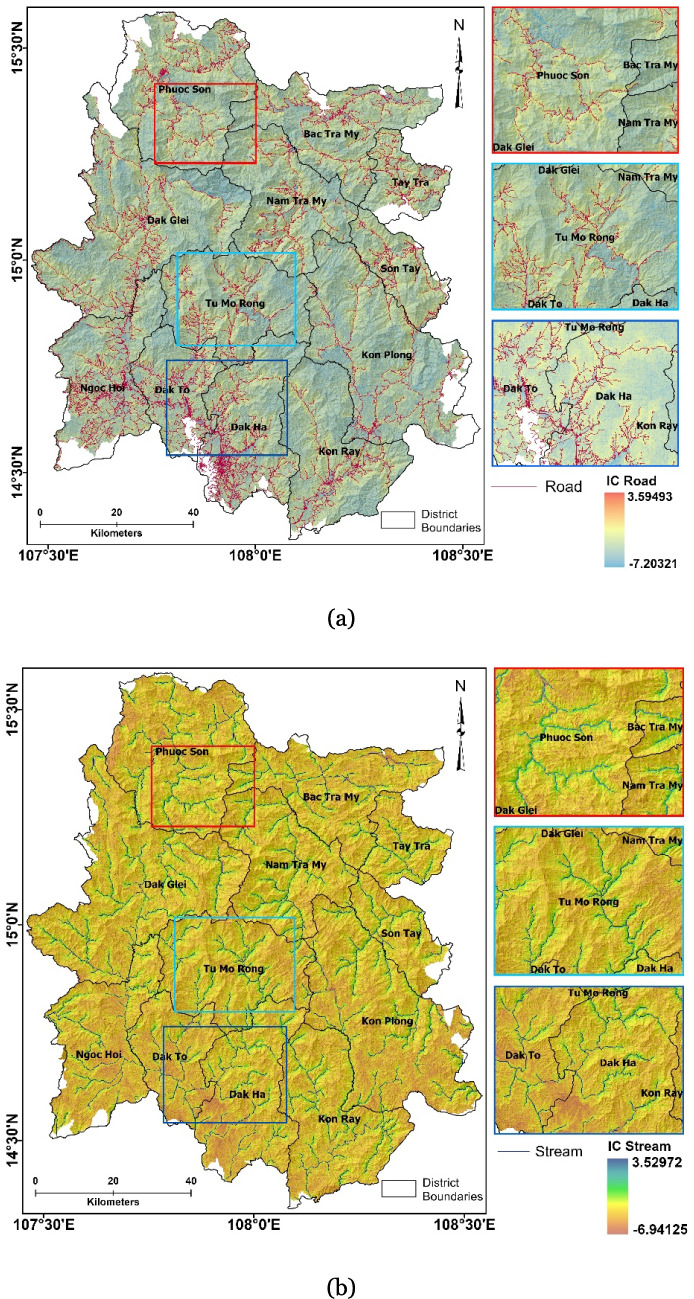
Maps showing the index of connectivity (IC) developed for roads (**a**) and streams (**b**). Different connectivity scenarios are highlighted in the panels to the right of each map, with their locations identified by the different colored boxes on the main map

The IC for road networks and river channels within the study area were analyzed, and the results are illustrated in Figs. 6 a and b, respectively. The topographic connectivity models developed in the study were utilized to quantify the landslide risk for roads and streams by integrating the IC information with the landslide hazard scenarios. The road networks have a high density in the southern part near the Dak Ha and Dak To districts in Kon Tum, while density is relatively low in the study area’s north, central, and east parts. The connectivity of the road network with the topography is well established, with IC_Road_ values ranging from + 3.59 (high) to −7.20 (low). Due to the dominance of high mountain topography for much of the study area, especially in the central and northern parts, roads passing through these regions are highly connected to the adjacent steep hillslopes, which manifests as high IC values in the model. Consequently, such high topographic connectivity increases the risk of landslides potentially impacting these roads. The southern part of the study area has the highest road density, resulting in greater connectivity to the topography. However, the relatively low elevation and flat terrain of the southern portion lessens the possibility of landslide initiations, resulting in a low risk for landslides despite high topographic connectivity. IC_Stream_ values ranged from + 3.52 (high) to −6.94 (low). Since rivers and creeks flow through the lowest elevations relative to their surroundings, the possible connectivity of these features with the adjacent topography encompasses large basin areas. The IC values are important to assess the risk of landslide-related damage to the streams and other critical facilitates situated along the stream networks due to the transport of debris material. The study area contains 53 critical infrastructural facilities of various capacities, including reservoirs, dams, and hydroelectric power stations along the stream networks. Increased topographic connectivity between areas highly susceptible to landslides and critical infrastructure such as dams, reservoirs, and hydroelectric facilities directly escalates the risk of landslide-induced damage to these structures.

### Landslide hazard and risk estimation

**Fig. 7:**
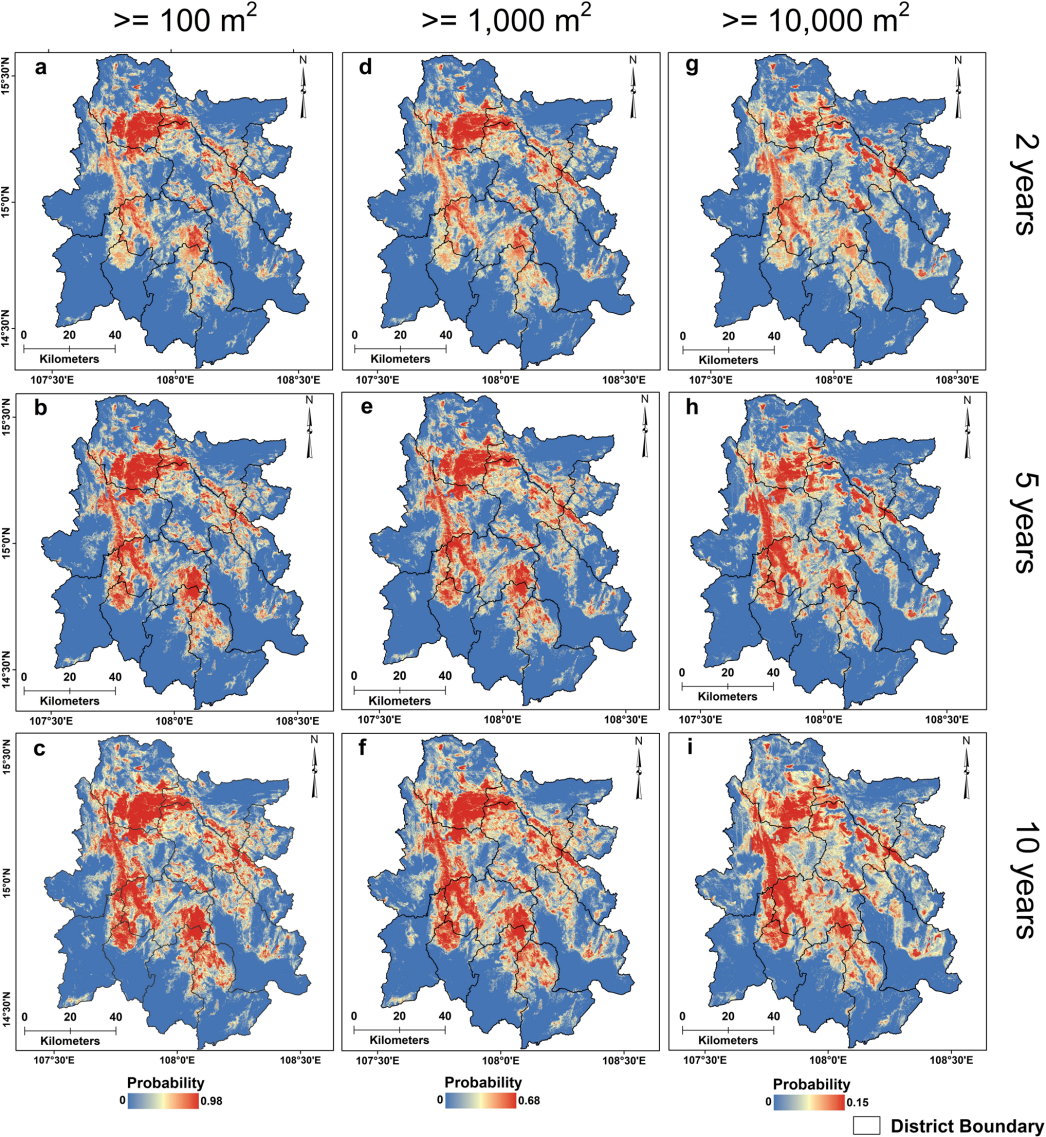
Maps showing the different probabilistic landslide hazard scenarios for landslide size exceeding 100 m^2^ (**a**-**c**), 1,000 m^2^ (**d**-**f**), and 10,000 m^2^ (**g**-**i**) occurring in the next two, five, and ten years in the study area

**Fig. 8:**
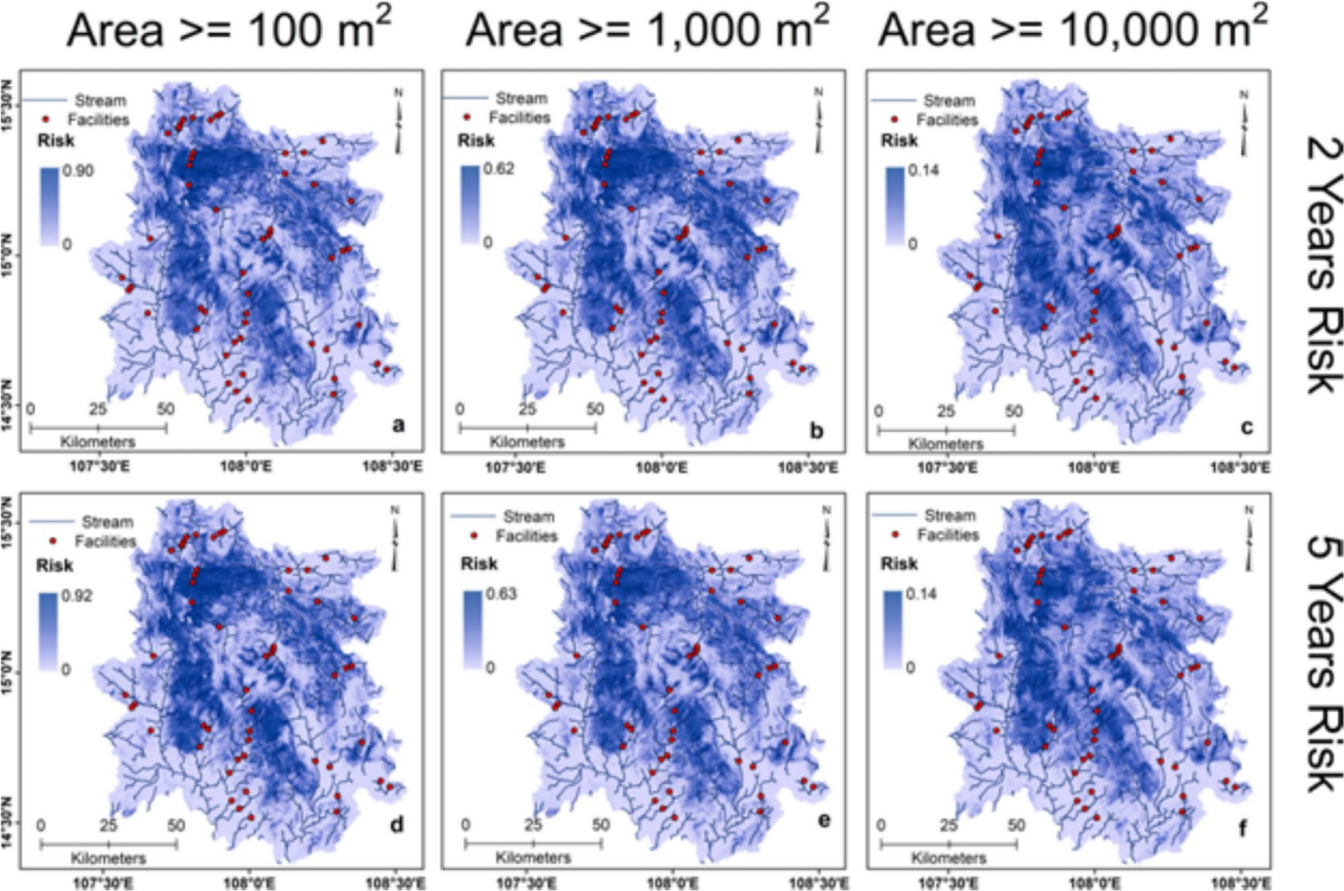
Maps showing quantified landslide risk scenarios of streams in the next two and five years from landslides of different sizes in the study area. Red dots are the critical infrastructural facilities, including dams, reservoirs, and hydroelectric power stations sited along stream channels

**Fig. 9:**
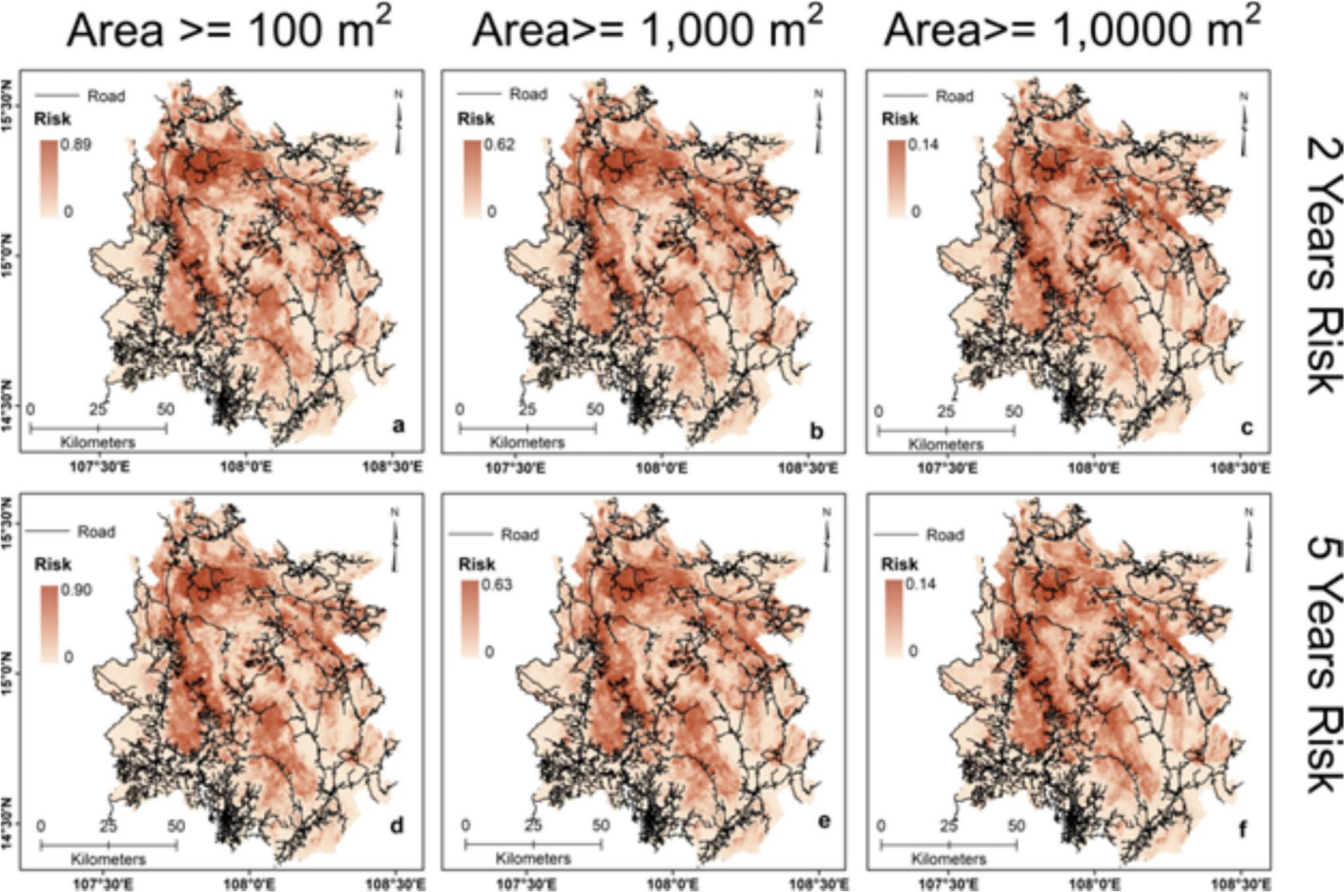
Maps showing quantified landslide risk scenarios of roads in the next two and five years from landslides of different sizes in the study area

Landslide hazard models were developed, integrating landslides' spatial, temporal, and size probabilities in the study area. A total of nine landslide hazard maps were generated, estimating the probability of occurrence of landslides of a size greater than 100 m^2^, 1,000 m^2^, and 10,000 m^2^ in the next two, five, and ten years for the study area. In general, the results show a high hazard probability for landslides having an area ≥ 100 m^2^ (0- 0.98) (Fig. 7 a-c), moderate for landslides with an area ≥ 1,000 m^2^ (0–0.68) (Fig. 7 d-f), and low for landslides larger than 10,000 m^2^ area (0–0.15) (Fig. 7 g-i) for two, five, and ten-year intervals. The probability of landslide hazard decreases with increased landslide size and rises with increased time for a constant landslide size. Every district in the study area has a high vulnerability to landslide hazards. However, the landslide hazard probability is very high in Phuoc Son, Nam Tra My, Dak Glei, and Tu Mo Rong districts and high in Kon Ray and Son Tay districts. Generally, the study area's southern and southwestern parts are largely low in landslide hazards. The landslide risk assessment was conducted by integrating the landslide hazard probability with the topographic connectivity index, irrespective of the size of the landslides. Figure 8 (a-f) shows the landslide risk obtained from local stream channels for landslide sizes ≥ 100 m^2^, ≥ 1,000 m^2^, and ≥ 10,000 m^2^ in the next two and five years. Landslide risk for streams is modeled to increase from 0.90 to 0.92 for landslides ≥ 100 m^2^ (Fig. 8a and d), from 0.62 to 0.63 for landslides ≥ 1,000 m^2^ (Fig. 8b and e), and risk remains almost same for landslides ≥ 10,000 m^2^ at 0.14 in the next five years (Fig. 8c and f). Figure 9 (a-f) illustrates landslide risk from landslide sizes ≥ 100 m^2^, ≥ 1,000 m^2^, and ≥ 10,000 m^2^ in the next two and five years for the roads. As with the landslide hazard, the risk decreases as the size increases and with increasing time. Landslide risk for the roads increases from 0.89 to 0.90 for landslides ≥ 100 m^2^ (Fig. 9a and d), from 0.62 to 0.63 for landslides ≥ 1,000 m^2^ (Fig. 9d and e), and risk remains almost constant for landslides ≥ 10,000 m^2^ at 0.14 in the next five years (Fig. 9c and f). Roads in the study area’s northern and central parts are at high risk of landslide-related damage. However, roads in the southern part near the study area boundary are at much lower risk. Landslide risk for both streams and roads in most parts of the study area achieve apex probabilities in the next five years' time and show little increase in the next 10 years (Supplementary Fig. C.4). Streams and other critical infrastructural facilities such as dams, reservoirs, and hydroelectric power stations, especially those located in the central and northern region of the study area, are at high risk of landslides. Due to the strong topographical connectivity between streams, critical infrastructures, and high landslide-prone zones, debris materials can readily be transported to these channels and structures during a landslide event, resulting in significant damage.

## Discussion

Regional-scale landslide hazard was estimated in this study by integrating probabilities of landslide occurrence in terms of time, space, and size-magnitude. Building on this, we proposed a practical methodology for quantifying landslide risk in data-sparse environments. While many researchers conceptualize landslide risk as the expected loss of lives and economic value, this study advances a modified approach that unifies spatial, temporal, and size-magnitude landslide probabilities with the topographic connectivity of vulnerable assets—such as roads, railways, buildings, and navigable rivers—to compute landslide risk. The core premise of this framework is that objects physically connected to susceptible parts of the landscape are at heightened risk of landslide-related damage. For example, if sections of a road or river are well connected to landslide-prone topography, those sections are classified as high-risk zones.

A significant strength of this approach is its simplicity. The model requires minimal input data, much of which is publicly available. It can be implemented using any open-source GIS platform, making it highly suited to regions with limited data and resources.

Given the absence of reliable pre-existing landslide data in the study area, inventories from Typhoon Ketsana, Tropical Storm Podul, and Typhoon Molave (2009, 2013, and 2020, respectively) were used to develop the landslide susceptibility models. These events were assumed to provide a representative snapshot of the current landslide regime, with the resulting susceptibility models applied to predict both present and future landslide occurrences. However, we acknowledge that this assumption may be challenged if future catastrophic storms trigger landslides in areas with little prior activity. Consequently, regular updates to the landslide hazard model are essential to improve monitoring efforts and inform more effective mitigation strategies as new data become available.

### Landslide susceptibility models

Landslide hazards and risk assessments at medium to regional scales rely heavily on the accuracy of the estimation of landslide susceptibility. In this study, models were developed for three landslide size categories (≥ 100 m^2^, ≥ 1,000 m^2^, and ≥ 10,000 m^2^) using the combined inventories from Ketsana, Podul, and Molave. Ideally, hazard estimation would be enhanced by calculating distinct temporal probabilities for each landslide size. However, such an analysis requires robust historical records of landslide events—data unavailable for this study area. Therefore, the temporal probability of specific landslide sizes was not integrated into the susceptibility models.

The models, built using a Random Forest algorithm, demonstrated high accuracy, sensitivity, and AUC scores on test data (Table 2), underscoring their robustness and predictive capability. Random Forest, an ensemble machine learning algorithm, constructs multiple decision trees and aggregates their predictions. It typically yields models with low bias and variance—well-suited for detecting complex patterns and ensuring reliable predictions on unseen data.

### Temporal probability estimation

Rainfall is the primary landslide trigger in central Vietnam. We used district-level records of rainfall-triggered landslides over the past six years to estimate the temporal probability. Unlike some previous studies (e.g., Jaiswal & Van Westen 2009; Tien Bui et al. 2012), which estimate temporal probability based on empirical rainfall-landslide relationships, this study calculated exceedance probabilities directly using documented landslide counts and applied a Poisson distribution model. The temporal probability thus represents the likelihood of at least one landslide event occurring within a specific area over a defined period.

Our analysis found that probability increases with time, reaching a peak after about eight years, suggesting that all districts will likely experience at least one landslide within that timeframe. However, significant variation was observed across districts. For example, Nam Tra My district has a 100% probability of experiencing a landslide within two years, whereas Tu Mo Rong requires six years to reach the same probability. It is important to note that these probabilities are conservative because the model is based only on documented events, and many smaller or undocumented landslides are likely missing. To improve temporal probability estimates, future work should focus on expanding historical landslide datasets and implementing more comprehensive documentation practices.

### Landslide size probability

The destructiveness or damage caused by landslides is conceptualized as the magnitude of landslides (Hungr 1997; Guzzetti et al. 1999). The severity of damage or destructiveness of a landslide largely depends on several factors, such as its volume, transport velocity, and kinetic energy. For a regional-scale study, acquiring and integrating such information into a hazard model is arduous. Accurately determining the volume of landslides for large events, such as the ones addressed in this study, where several thousands of landslides were triggered, poses a significant challenge. Moreover, comprehensive inventorying of many landslides would typically rely on satellite imagery, which presents challenges in accurately calculating their volume. Therefore, landslide areas are often used as a proxy for volumes, assuming that larger areas correspond to higher volumetric yield and, consequently, greater potential for property destruction or damage than smaller landslides. Assuming that landslide size is a proxy for potential destructiveness, the hazard model utilizes the probabilities of sizes exceeding 100 m^2^, 1,000 m^2^, or 10,000 m^2^ from the combined inventories. By utilizing a combined inventory, the study aimed to capture the average size of landslides in the area over time, assuming this average reflects the probability distribution of landslide sizes in the region and that future landslides will exhibit similar characteristics.

### Landslide hazard assessment

The landslide hazard model assumes the conditional independence of landslides’ spatial, temporal, and size probabilities. Spatial probability was calculated pixel-by-pixel, while temporal probability was estimated at the district level based on the frequency of landslide occurrences in the past six years, all triggered by rainfall. It is important to note that the developed landslide susceptibility model incorporates annual average rainfall as a variable, which may not directly correlate with the frequency of landslide occurrences, as landslides can occur in areas with both high and low rainfall. Additionally, parts of certain districts with high landslide susceptibility, such as To Mo Rong, Dak Ha, and Dak To, exhibit lower temporal landslide probabilities. Hence, we infer that spatial and temporal landslide probabilities are independent processes in this study. Similarly, the analysis of size probabilities for the three events indicates that landslide size is also independent of time and spatial events. The landslide hazard probability in central Vietnam generally decreases as the landslide size increases while the temporal probability remains constant. Larger landslides (e.g., 10,000 m^2^) are less frequent than smaller ones (100 m^2^), so the hazard probability associated with large landslides is relatively low. Conversely, the probability of landslide hazard increases with an increase in the time while keeping the landslide size constant. As the probability of one or more landslides occurring increases over time, the landslide hazard probability for the next ten years is higher than that for the next two years.

### Landslide risk framework

Our risk framework combines topographic connectivity with hazard probabilities to evaluate regional-scale landslide risk. Due to the challenges of developing a separate runout model at this scale, the focus was on assessing risk to roads and stream networks using connectivity metrics. The rationale is that well-connected segments exposed to highly susceptible topography face a greater risk of landslide-induced damage. It is important to note that the quantified landslide risk model does not provide an exact probability of landslide risk, as the IC model values were originally computed on a logarithmic scale and later normalized to a 0–1 scale for integration with landslide probabilities. However, the quantified risk scale functions similarly to a probability scale, where values closer to 1 indicate higher risk and values closer to 0 indicate lower landslide risk. Since estimating monetary losses associated with landslides was not feasible due to unavailability of data, integrating the connectivity model with landslide hazard probabilities proved to be an effective approach for understanding the spatial–temporal risk of these assets in a data-scarce environment. Risk assessment for stream networks aimed to delineate potential locales susceptible to large influxes of landslide-delivered sediment, which could adversely affect the carrying capacity of the receiving streams and increase the region’s vulnerability to flooding, another significant hazard in the area. Moreover, numerous dams, water reservoirs and hydroelectric plants are located along these streams could also be impacted by sediment influx, rendering them operationally vulnerable or even susceptible to destruction from direct landslides. Hence, understanding the topographic connectivity of these rivers is crucial for designing preventive measures to minimize landslide-related damages. Similarly, road networks are highly susceptible to slope failures, and the risk model facilitated the identification of highly vulnerable sections, allowing for allocating resources toward implementing suitable mitigation measures. Although a similar risk model could have been developed for individual buildings or other assets to estimate potential danger, the lack of relevant information limited such analysis in the present study. Nevertheless, it is important to reiterate the necessity of updating landslide risk models with the availability of additional data to ensure their efficacy in mitigating landslides’ adverse effects on society and regional economies.

## Conclusion

Considering the growing occurrence and severity of landslides, comprehending the dynamics of landslide events and identifying potential hazardous zones is of utmost importance. This knowledge is vital for planners, disaster management authorities, decision-makers, and individual landowners in central Vietnam. The study successfully conducted a probabilistic landslide hazard assessment, which was implemented using an open-source platform. Furthermore, a novel approach has been proposed for quantifying landslide risk in data-sparse environments.

The probabilistic landslide hazard model developed in this study follows the framework proposed by Varnes (1984) and Guzzetti et al. (1999). The model incorporates the joint probability of landslides’ spatial, temporal, and size probabilities. Landslide susceptibility analysis—known as spatial probability—was performed for landslide sizes ≥ 100 m^2^, ≥ 1,000 m^2^, and ≥ 10,000 m^2^ using three inventories of landslides triggered by Typhoon Ketsana, Tropical Storm Podul, and Typhoon Molave in central Vietnam in 2009, 2013, and 2020, respectively. Mean annual rainfall and elevation are the most influential variables shaping the spatial distribution of landslides. Owing to limited data, the temporal probability estimation of this study is based on landslide occurrence data from the past six years. The size probability assessment, a proxy for landslide magnitude, was based on a size-frequency analysis of the three landslide inventories. Nevertheless, the accuracy and robustness of the analyses concerning spatial, temporal, and size probabilities would significantly benefit from more accurate and comprehensive historical landslide data.

This study developed nine probabilistic landslide hazard scenarios by integrating spatial, temporal, and size probabilities. Results show that hazard probability decreases with increasing landslide size and increases over longer forecast periods. Small landslides (≥ 100 m^2^) were the highest hazard, particularly in districts such as Phuoc Son, Nam Tra My, and Tu Mo Rong. This study also proposed a novel methodology for landslide risk assessment by integrating probabilistic landslide hazard with an index of connectivity. While landslide risk can be assessed for various elements depending on planning needs, this study focused on two highly vulnerable components: road and stream networks. Nine risk scenarios were developed for these networks, projecting potential impacts over the next 2, 5, and 10 years for landslide sizes ≥ 100 m^2^, ≥ 1,000 m^2^, and ≥ 10,000 m^2^. The results show that risk increases with time and is highest for smaller landslides, with stream networks exhibiting more consistent risk exposure than roads. This approach provides a flexible and scalable method for quantifying landslide risk, especially in data-scarce environments. The hazard and risk outputs generated in this study offer critical insights to support sustainable land use planning and infrastructure development in the landslide-prone regions of central Vietnam.

## Supplementary Information

Below is the link to the electronic supplementary material.

Supplementary file1 (PDF 62468 KB)

## Data Availability

Data will be made available upon request.

## References

[CR1] Aksha SK, Resler LM, Juran L, Carstensen LW Jr (2020) A geospatial analysis of multi-hazard risk in Dharan. Nepal Geomatics, Natural Hazards and Risk 11(1):88–111

[CR2] Arrogante-Funes P, Bruzón AG, Arrogante-Funes F, Ramos-Bernal RN, & Vázquez-Jiménez R (2021). Integration of vulnerability and hazard factors for landslide risk assessment. International journal of environmental research and public health, 18(22):11987 10.3390/ijerph182211987 PMC8623781 34831741

[CR3] Banshtu RS, Versain LD, Pandey DD (2020) Risk assessment using quantitative approach: central Himalayas, Kullu, Himachal Pradesh, India. Arab J Geosci 13:1–11

[CR4] Borselli L, Cassi P, Torri D (2008) Prolegomena to sediment and flow connectivity in the landscape: a GIS and field numerical assessment. CATENA 75(3):268–277

[CR5] Bui DT, Tsangaratos P, Nguyen VT, Van Liem N, Trinh PT (2020) Comparing the prediction performance of a deep learning neural network model with conventional machine learning models in landslide susceptibility assessment. CATENA 188:104–426

[CR6] Carter A, Roques D, Bristow CS (2000) Denudation history of onshore central Vietnam: constraints on the Cenozoic evolution of the western margin of the South China Sea. Tectonophysics 322(3–4):265–277

[CR7] Cavalli M, Trevisani S, Comiti F, Marchi L (2013) Geomorphometric assessment of spatial sediment connectivity in small Alpine catchments. Geomorphology 188:31–41

[CR8] Coe JA, Michael JA, Crovelli RA, Savage WZ, Laprade WT, Nashem WD (2004) Probabilistic assessment of precipitation-triggered landslides using historical records of landslide occurrence, Seattle. Washington Environmental & Engineering Geoscience 10(2):103–122

[CR9] Crema S, Cavalli M (2018) Sedinconnect: a stand-alone, free and open source tool for the assessment of sediment connectivity. Comput Geosci 111:39–45

[CR10] Crovelli RA (2000) Probability models for estimation of number and costs of landslides. foot (ft) 25:0–3048

[CR11] Das Iswar, et al (2011) Probabilistic landslide hazard assessment using homogeneous susceptible units (HSU) along a national highway corridor in the northern Himalayas, India. Landslides 8(3): 293–308

[CR12] Das R, Tien PV, Wegmann KW, Chakraborty M (2024) Machine learning-based assessment of regional-scale variation of landslide susceptibility in central Vietnam. PLoS One 19(10):e0308494 39453912 10.1371/journal.pone.0308494 PMC11508152

[CR13] Dikshit A, et al (2020) Temporal probability assessment and its use in landslide susceptibility mapping for eastern Bhutan. Water 12(1):267

[CR14] Duc DM, Khang DQ, Duc DM, Ngoc DM, Quynh DT, Thuy DT, Giang NK, Van Tien P, Ha NH (2020) Analysis and modeling of a landslide-induced tsunami-like wave across the Truong river in Quang Nam province, Vietnam. Landslides 17:2329–2341

[CR15] Fangqian W, Jinhai Y, DinhLuyen N, Wei J (2020) Precambrian crust components and its tectonic evolution of the Kontum massif, central Vietnam. Geological Journal of China Universities 26(2):161

[CR16] Froude MJ, Petley DN (2018) Global fatal landslide occurrence from 2004 to 2016. Nat Hazards Earth Syst Sci 18(8):2161–2181

[CR17] Fu Sheng, et al (2020) Landslide hazard probability and risk assessment at the community level: A case of western Hubei, China. Nat Hazards Earth Syst Sci 20(2):581–601

[CR18] Funk C, Peterson P, Landsfeld M, Pedreros D, Verdin J, Shukla S, Husak G, Rowland J, Harrison L, Hoell A, Michaelsen J (2015) The climate hazards infrared precipitation with stations—a new environmental record for monitoring extremes. Sci Data 2(1):1–21 10.1038/sdata.2015.66 PMC4672685 26646728

[CR19] Ghosh S, Van Westen CJ, Carranza EJ, Ghoshal TB, Sarkar NK, Surendranath M (2009) A quantitative approach for improving the BIS (Indian) method of medium-scale landslide susceptibility. J Geol Soc India 74:625–638

[CR20] Ghosh S, van Westen CJ, Carranza EJM et al (2012) Integrating spatial, temporal, and magnitude probabilities for medium-scale landslide risk analysis in Darjeeling Himalayas, India. Landslides 9:371–384

[CR21] Guzzetti F, Carrara A, Cardinali M, Reichenbach P (1999) Landslide hazard evaluation: a review of current techniques and their application in a multi-scale study, Central Italy. Geomorphology 31(1–4):181–216

[CR22] Guzzetti F, Carrara A, Cardinali M, Reichenbach P (2005) Probabilistic landslide hazard assessment at the basin scale. Geomorphology 72(1–4):272–299

[CR23] Hervás J, Bobrowsky P (2009) Mapping: inventories, susceptibility, hazard and risk. Landslides–disaster risk reduction pp 321–349

[CR24] Hùng PV, Dũng NV (2013) Risk warning landslide in the mountainous districts of Quang Ngai province. Vietnam J Earth Sci 35(2):107–119

[CR25] Hungr O (1997) Some methods of landslide hazard intensity mapping. In: Landslide risk assessment. Routledge, p 215–226

[CR26] Jaiswal P, van Westen CJ (2009) Estimating temporal probability for landslide initiation along transportation routes based on rainfall thresholds. Geomorphology 112(1–2):96–105

[CR27] Jaiswal P, van Westen CJ, Jetten V (2010) Quantitative landslide hazard assessment along a transportation corridor in southern India. Eng Geol 116(3–4):236–250

[CR28] Jakob M (2022) Landslides in a changing climate. In: Landslide hazards, risks, and disasters. Elsevier, p 505–579

[CR29] Jiang W, Yu JH, Griffin WL, Wang F, Wang X, Pham T, Nguyen D (2022) Where did the Kontum Massif in central Vietnam come from? Precambr Res 377:106–725

[CR30] Le Hong L, Van P (2016) Mapping of large scale landslide topographic area by aerial photograph interpretation and possibilities for application to risk assessment for the Ho Chi Minh route Vietnam. Transaction, Japanese Geomorphological Union 37(1):97–118

[CR31] Malamud BD, Turcotte DL, Guzzetti F, Reichenbach P (2004) Landslide inventories and their statistical properties. Earth Surf Process Landforms 29(6):687–711

[CR32] Meinhardt M, Fink M, Tu¨nschel H (2015) Landslide susceptibility analysis in central Vietnam based on an incomplete landslide inventory: comparison of a new method to calculate weighting factors by means of bivariate statistics. Geomorphology 234:80–97

[CR33] Metternicht G, Hurni L, Gogu R (2005) Remote sensing of landslides: an analysis of the potential contribution to geo-spatial systems for hazard assessment in mountainous environments. Remote Sens Environ 98(2–3):284–303

[CR34] Nakano N, Osanai Y, Owada M, Binh P, Hokada T, Kaiden H, Bui VT (2021) Evolution of the Indochina block from its formation to amalgamation with Asia: constraints from protoliths in the Kontum Massif. Vietnam. Gondwana Res 90:47–62

[CR35] NASA JPL (2020) NASADEM Merged DEM Global 1 arc second V001. NASA EOSDIS Land Processes DAAC

[CR36] Nguyen CC, Vo P, Doan VL, Nguyen QB, Nguyen TC, Nguyen QD (2023) Assessment of the effects of rainfall frequency on landslide susceptibility mapping using AHP method: A case study for a mountainous Region in Central Vietnam. In: Progress in Landslide Research and Technology, Volume 1 Issue 2, 2022. Springer, p 87–98

[CR37] Osanai Y, Nakano N, Owada M, Nam TN, Toyoshima T, Tsunogae T, Binh P (2004) Permo-Triassic ultrahigh- temperature metamorphism in the Kontum massif, central Vietnam. J Mineral Petrol Sci 99(4):225–241

[CR38] Ram P, Gupta V (2021) Landslide hazard, vulnerability, and risk assessment (HVRA), Mussoorie township, lesser Himalaya, India. Environ Dev Sustain. 10.1007/s10668-021-01449-2

[CR39] Saleem J, Ahmad SS, Butt A (2020) Hazard risk assessment of landslide-prone sub-Himalayan region by employing geospatial modeling approach. Nat Hazards 102:1497–1514

[CR40] Tien Bui D, Pradhan B, Lofman O, Revhaug I (2012) Landslide susceptibility assessment in Vietnam using support vector machines, decision tree, and Naive Bayes Models. Mathematical problems in Engineering 2012

[CR41] Tien Bui D, Pradhan B, Lofman O et al (2013) Regional prediction of landslide hazard using probability analysis of intense rainfall in the Hoa Binh province, Vietnam. Nat Hazards 66:707–730. 10.1007/s11069-012-0510-0

[CR42] Tien PV, Sassa K, Takara K, Tam DM, Quang LH, Khang DQ, Luong LH, Loi DH (2016) The influence of rainfalls on the potential of landslide occurrence on Hai Van Mountain in Vietnam. In: Proceeding of the final SATREPS workshop on landslides, pp 112–121

[CR43] Van Den Eeckhaut M, Poesen J, Govers G, Verstraeten G, Demoulin A (2007) Characteristics of the size distribution of recent and historical landslides in a populated hilly region. Earth Planet Sci Lett 256(3–4):588–603

[CR44] Van Tien P, Luong LH, Duc DM, Trinh PT, Quynh DT, Lan NC, Thuy DT, Phi NQ, Cuong TQ, Dang K, Loi DH (2021) Rainfall-induced catastrophic landslide in Quang Tri Province: the deadliest single landslide event in Vietnam in 2020. Landslides. 10.1007/s10346-021-01664-y

[CR45] Van Tien P, Luong LH, Nhan TT, Duc DM, Quynh DT, Lan NC, Phi NQ, Hao DC, Ha NH, Thuy DT, Thao VB (2021b) Secondary processes associated with landslides in vietnam. In: Proceedings of the International Conference on Innovations for Sustainable and Responsible Mining: ISRM 2020-Volume 2, Springer, pp 192–209

[CR46] Van Westen C, Van Asch TW, Soeters R (2006) Landslide hazard and risk zonation—why is it still so difficult? Bull Eng Geol Environ 65:167–184

[CR47] Varnes DJ (1984) Landslide hazard zonation: a review of principles and practice.

[CR48] Yang H, Wei F, Ma Z, Guo H, Su P, & Zhang S (2020) Rainfall threshold for landslide activity in Dazhou, southwest China. Landslides, 17(1):61–77

[CR49] Zêzere JL, Reis E, Garcia R, Oliveira S, Rodrigues ML, Vieira G, and Ferreira AB (2004) Integration of spatial and temporal data for the definition of different landslide hazard scenarios in the area north of Lisbon (Portugal), Nat Hazards Earth Syst Sci 4:133–146. 10.5194/nhess-4-133-2004

