# Supplementary material for: Quantitative assessment of landslide hazard and risk at regional-scale: a case study from central Vietnam
Source: Environ Sci Pollut Res Int. 2025 Dec 18;32(56):30776–93. doi: 10.1007/s11356-025-37189-3 (PMC12804295; doi:10.1007/s11356-025-37189-3)
Supplement: Supplementary file 1 — Supplementary file1 (PDF 62468 KB) [file 11356_2025_37189_MOESM1_ESM.pdf]

Supplementary Materials for

Quantitative Assessment of Landslide Hazard and Risk at Regional-Scale: A case Study from Central Vietnam

Raja Das*, Pham Van Tein, Karl W. Wegmann

*Corresponding author. Email: rdas3@ncsu.edu

Appendix A. Supplementary Methods

Appendix A.1. Modeling algorithm

The construction of the landslide susceptibility models was based on the Random Forest (RF) algorithm, a robust ensemble machine learning method characterized by its utilization of multiple decision trees (DTs) in a non-parametric framework. Each decision tree within the RF model is composed of nodes representing the variables involved in the prediction, starting from a root node and branching out into decision nodes. These nodes are determined by the Gini Impurity measure, which assesses the likelihood of incorrect classification if an observation were randomly labeled, with preference given to variables with lower Gini impurity for positioning at the top of the tree. The branching continues until it reaches a point where no further splits are feasible, forming the terminal nodes.

The RF algorithm employs 'Bootstrap Aggregating' or 'Bagging,' a technique that creates a random subset of the training data with replacement for each DT, allowing for the same data point to be used in multiple trees. This process results in a collection of de-correlated decision trees that are trained simultaneously on these bootstrap samples. Typically, two-thirds of the data are used for tree construction, with the remaining one-third reserved for model validation through the calculation of the out-of-bag error rate.

Aggregation is a crucial step in the RF model, where the final prediction is derived from the majority vote (in classification tasks) or the average (in regression tasks) across all decision trees' outcomes. This aggregation of multiple 'weak learners' into a 'strong learner' significantly enhances model accuracy and reduces variance, addressing the overfitting issue commonly associated with single decision trees.

The RF model was developed using R statistical software, incorporating a five-fold cross-validation process repeated ten times to ensure reliability. The *Mtry* hyperparameter, which dictates the number of variables considered for splitting at each node, was optimized based on model accuracy, leading to the selection of an ideal *Mtry* value. The model consisted of 500 decision trees, balancing complexity and computational efficiency.

Appendix A.2. Developing Topographic Connectivity Index

Following is the formula to compute the IC of a landscape:

$$IC = \log_{10}\left(\frac{D_{up}}{D_{dn}}\right) = \log_{10}\left(\frac{\overline{WS}\sqrt{A}}{\sum_i \frac{d_i}{W_i S_i}}\right) \tag{A.1}$$

Where D_{up} is the upslope component and indicates downslope routing of sediment generated upslope, D_{dn} is the downslope component that refers to the flow length that a particle travels to reach the user specified targets (e.g., streams, road, building). $\overline{S}$ is the average slope gradient of the upslope contributing area (m/m), A is the upslope contributing area (m^2), $\overline{W}$ is the average weighting factor of the upslope contributing area, which is equivalent to the standardized measure of Roughness Index (RI) (Cavalli et al. 2013):

$$W = \left(1 - \frac{RI}{RI_{max}}\right) \tag{A.2}$$

where, RI_{Max} is the maximum value of RI in the study area.

RI is the measure of the standard deviation of the residual topography (Cavalli and Marchi 2008; Cavalli et al. 2008)computed as the difference between unsmoothed and smoothed DTMs that represents local scale obstacles for sediment transportation to the nearest target. In the downslope component (D_{dn}), d_i is the flow path length along the i th cell according to the steepest downslope direction (m), and W_i and S_i are the weighting factor and the slope gradient of the ith cell, respectively.

References

Cavalli, M., Marchi, L., 2008. Characterisation of the surface morphology of an alpine alluvial fan using airborne lidar. Natural Hazards and Earth System Sciences 8, 323–333.

Cavalli, M., Tarolli, P., Marchi, L., Dalla Fontana, G., 2008. The effectiveness of airborne lidar data in the recognition of channel-bed morphology. Catena 73, 249–260.

Cavalli, M., Trevisani, S., Comiti, F., Marchi, L., 2013. Geomorphometric assessment of spatial sediment connectivity in small alpine catchments. Geomorphology 188, 31–41.

Appendix B. Supplementary Table

Table B.1: Reclassified lithological units in the study area

Group	Description	Area(%)
1	Mafic Metamorphic Rocks with quartz-poor component	46.9
2	Igneous - Instrusive Acid-to-Neutral Rocks	26.6
3	Igneous - Intrusive Mafic-to-Ultramafic Rocks	0.8
4	Felsic Metamorphic Rocks with quartz-rich component	16.1
5	Igneous- Extrusive Mafic-to-Ultramafic Rocks	5.0
6	Sedimentary Clastic Rocks	2.8
7	Quaternary Sediments	1.8

Appendix C. Supplementary Figures

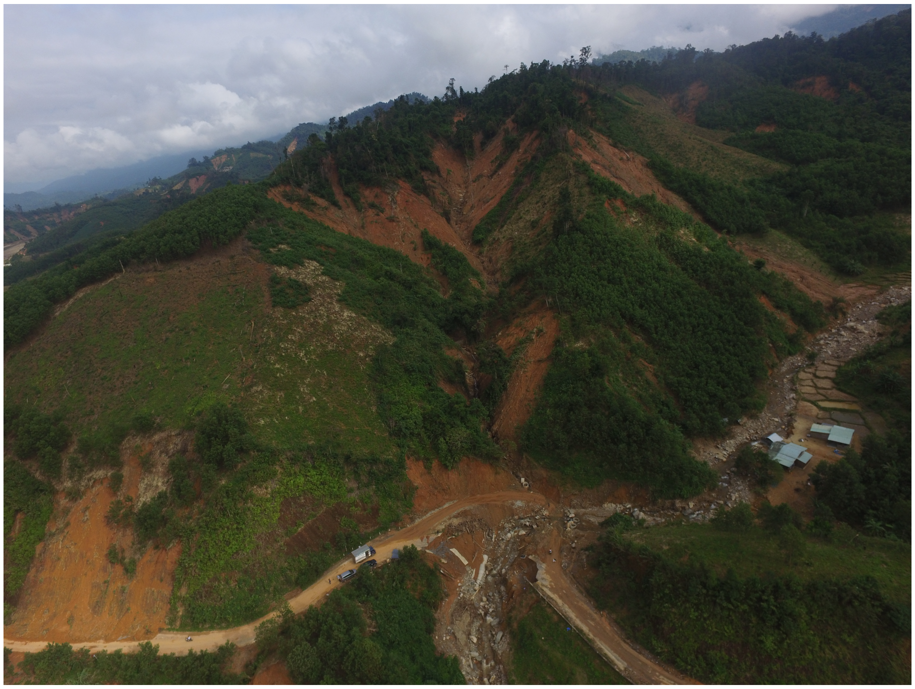

(a)

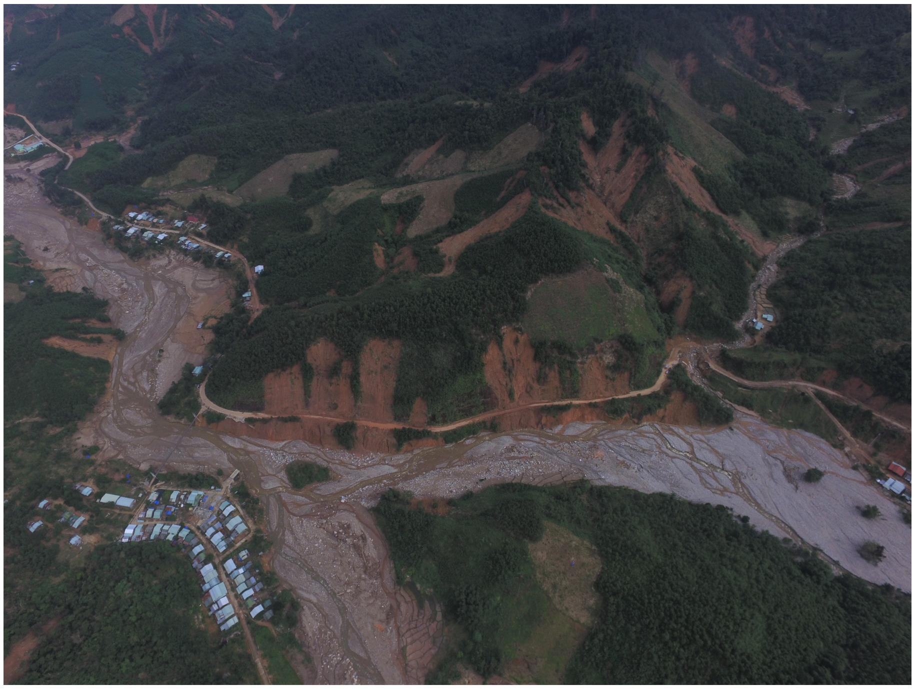

(b)

Figure C.1: Field photos of landslides triggered by Typhoon Molave in 2020 captured by UAV in Quang Nam Province.

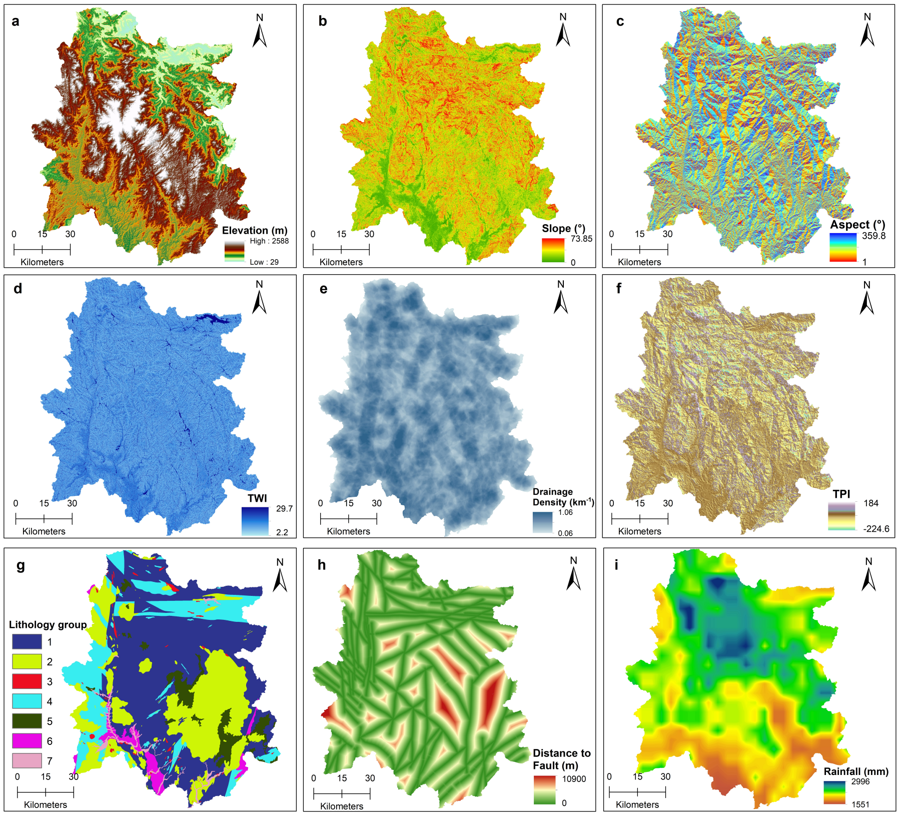

Figure C.2: Maps of the different explanatory variables used for building the Random forest model. (a) Elevation,(b) Slope angle, (c) Slope aspect, (d) Topographic Wetness Index (TWI), (e) Drainage Density, (f) Topographic Position Index (TPI), (g) Rock types (lithology group description in Table B.1), (h) Distance from fault, and (i) Annual average rainfall from 1990 to 2020 for the months between July and November.

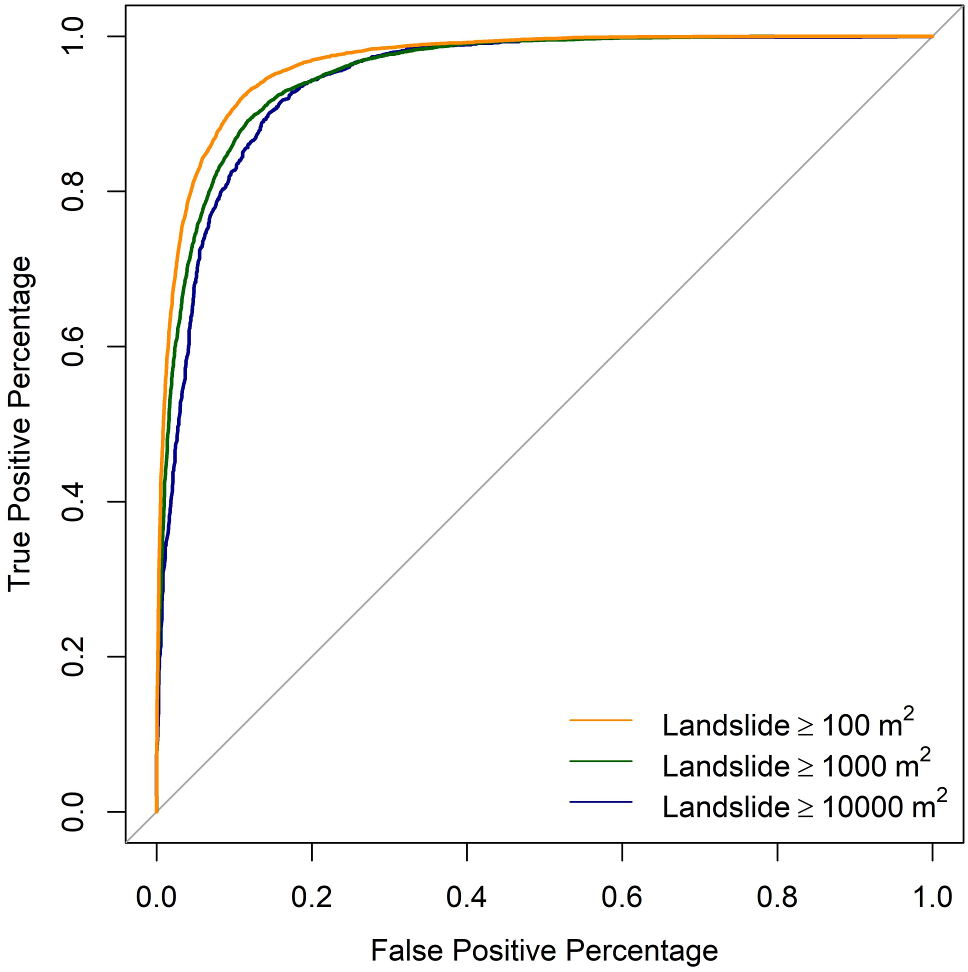

Figure C.3: AUC plots of landslide susceptibility models for landslide size ≥ 100 m^2, ≥ 1,000 m^2, and ≥ 10,000 m^2 marked in orange,green and blue colors, respectively.

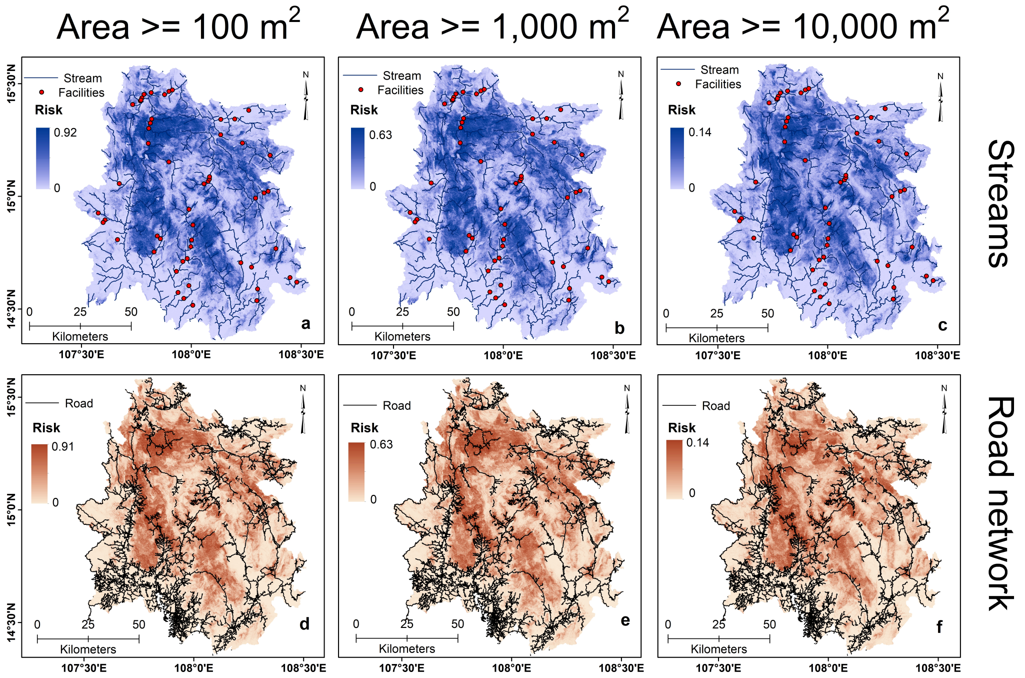

Figure C.4: Map showing landslide risk scenarios for 10 years time for streams and roads from landslides of different sizes.Red dots are the critical infrastructural facilities including dams, reservoirs, and hydroelectric power stations located along the stream channels.
